# Supplementary material for: A New Glutathione-Cleavable Theranostic for Photodynamic Therapy Based on Bacteriochlorin e and Styrylnaphthalimide Derivatives
Source: Biosensors (Basel). 2022 Dec 8;12(12):1149. doi: 10.3390/bios12121149 (PMC9775103; doi:10.3390/bios12121149)
Supplement: Supplementary file 1 [file biosensors-12-01149-s001.zip › biosensors-2048547-supplementary.pdf]

biosensors

Electronic Supplementary Information

A new glutathione-cleavable theranostic for photodynamic therapy based on bacteriochlorin e and styrylnaphthalimide derivatives

Marina A. Pavlova, Pavel A. Panchenko, Ekaterina A. Alekhina, Anastasia A. Ignatova, Anna D. Plyutinskaya, Dmitriy A. Pritmov, Mikhail A. Grin, Alexey V. Feofanov, Olga A. Fedorova

CONTENTS

1. Synthesis of conjugate BChl-S-S-NI……………………………………………………………………………S2
2. ^{1}H and ^{13}C NMR spectra of BChl-S-S-NI (S2, S3)……………………………………………………… S6
3. Changes in the absorption spectra of mixed solutions containing photosensitizer and DPBF (S4)……………………………………………………………………………S7
4. Optical spectra (S5-S6)……………………………………………………………………………S8
5. Confocal fluorescent images (S7, S8)……………………………………………………………………………S9

Synthesis of the compounds

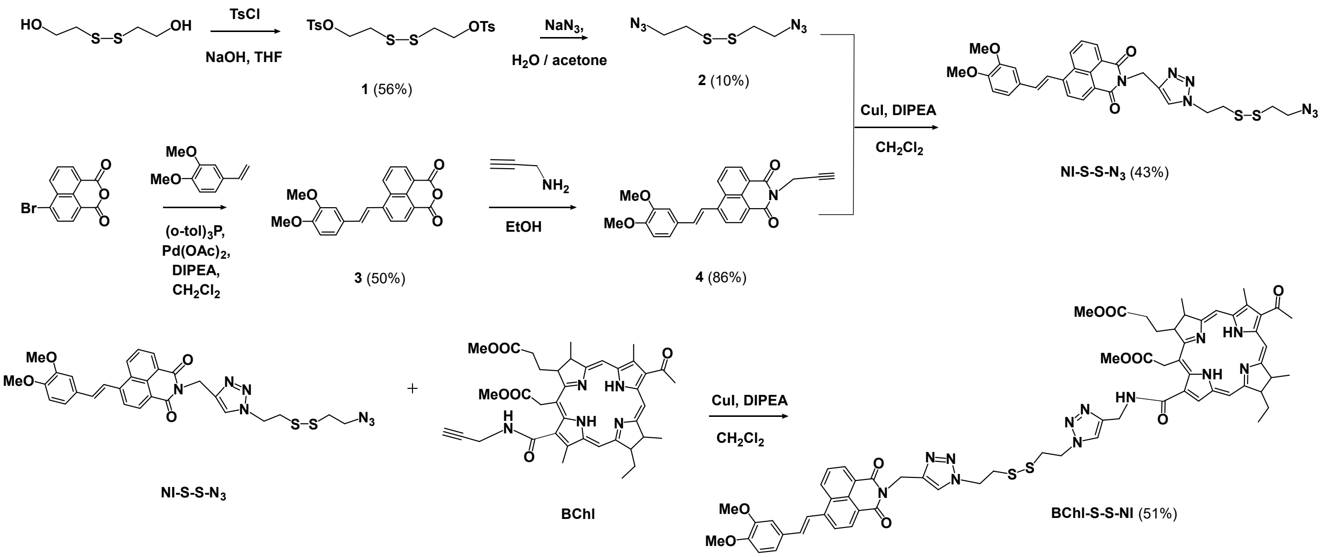

Figure S1. Synthetic scheme.

Apparatus.

^{1}H and ^{13}C NMR spectra were recorded on an Avance 400 (Bruker) and Inova 400 (Agilent) spectrometers operating at 400.13 MHz (for ^{1}H) and 100.60 MHz (for ^{13}C). The chemical shifts were determined with an accuracy of 0.01 ppm relative to residual solvent signals and translated to the internal standard (TMS), coupling constants were measured with an accuracy of 0.1 Hz. The assignment of ^{1}H and ^{13}C signals is based on 2D NMR experiments (HMBC, HSQC, ^{1}H COSY), which were performed using standard pulse sequences from the Bruker and Agilent library. The numbering of carbon atoms in the naphthalimide and bacteriochlorin fragments used for the description of the ^{1}H NMR spectra is shown on the corresponding structures before the description of the synthesis. In the case of conjugate BChl –S –S–NI, carbon atoms of naphthalimide are marked with a prime. Melting points were measured on Melt-temp melting point electrothermal apparatus and were uncorrected. The reaction course and purity of the final products was followed by TLC on silica gel (DC-Alufolien Kieselgel 60 F_{254}, Sigma-Aldrich) and aluminum oxide (Aluminium oxide 60 F_{254}, neutral, Merck). Flash column chromatography was conducted over silica gel (Kieselgel, 40-60 µm, Acros Organics) or aluminium oxide neutral (Brockmann I, 50-200 µm, Acros Organics) using preparative low pressure chromatograph Isolera Prime (Biotage). Preparative TLC was performed on silica gel 60 (Merck) using 20×20 cm plates with a layer thickness of 1 mm. Electron impact (EI) (70 eV) mass spectra were obtained from Finnigan Polaris Q instrument (ion-trap) in standard conditions. LC-ESI-MS analyses were performed on a Shimadzu LCMS-2020, using acetonitrile (Panreac, HPLC-gradient grade) as the mobile phase. The mass-spectra of BChl–S–S–NI obtained by MALDI method on MALDI-TOF spectrometer Shimadzu AXIMA Confidence using α-cyano-4-hydroxycinnamic acid as matrix. Elemental analyses were carried out in the Microanalysis Laboratory of the A.N. Nesmeyanov Institute of Organoelement Compounds. Starting compounds and reagents were obtained from commercial sources (Sigma Aldrich, Merck) and were used without any further purification

Synthetic procedures and characterization data.

2,2'-disulfanediylbis(ethane-2,1-diyl) bis(4-methylbenzenesulfonate) (1). A stirring solition of 4g (3.17 ml, 25.9 mmol) 2-hydroxyethyldisulfide in 17 ml THF was cooled to 0˚C at the ice-bath. Then a solution of 3.63 g (90.8 mmol) NaOH was added dropwise not allowing the mixture to warm up. In the same way a solution of 10.85g (56.9 mmol) p-toluenesulfonyl chloride in 25.5 ml THF was added. Reaction mixture was stirred at 0 °C during 1h and then 1h at room temperature. After, the reaction mass is poured into 62 ml of 10% (mass.) HCl solution and cooled. The product was filtered off, washed on the filter with distilled water and dried. Yield 6.72 g (56%). M.p. 54-56ºC. ^{1}H NMR (400.13MHz, $CDCl_3$, 24˚C): δ = 2.46 (s, 6H, 2×CH_3), 2.85 (t, 4H, *J* = 6.5, 2×CH_2), 4.21 (t, 4H, *J* = 6.5, 2×CH_2), 7.36 (d, 2H, *J* = 7.3, 2H-aryl), 7.80 (d, 2H, *J* = 7.3, 2H-aryl).

1,2-bis(2-azidoethyl)disulfane (2). Compound **1** 2.5 g (5.4 mmol) was dissolved in 62.5 ml of acetone an argon atmosphere. A solution of 1.788 g (27.5 mmol) NaN_3 in H_2O (38 ml) was added. A reaction mixture was stirred for 15h at 80˚C. Then acetone was removed in vacuum and resulting mixture was extracted with diethyl ether (3×30 ml). The organic layer was dried over anhydrous sodium sulfate, filtered and removed by reduced pressure. The crude product was purified by flesh-chromatography (Al_2O_3, eluent ethyl petroleum ether:acetate (5:1, v/v) to obtain the desired product **2** as a colourless viscous liquid (0.1095g, yield 10%). ^{1}H NMR (400.13 MHz, $CDCl_3$, 19˚C): δ = 2.88 (t, 4H, *J* = 6.7, 2×CH_2), 3.61 (t, 4H, *J* = 6.7, 2×CH_2).

(E)-6-(3,4-dimethoxystyryl)benzo[de]isochromene-1,3-dione (3). A mixture of 4-bromonaphthalic anhydride (0.25 g, 0.9 mmol), 3,4-dimethoxystyrene (0.160 ml, 1.08 mmol), $Pd(OAc)_2$ (2.4 mg, 0.01 mmol), $(o\text{-tol})_3P$ (15.0 mg, 0.05 mmol) and 1.5 ml (8.61 mmol) DIPEA in 10 ml DMF was stirred at 105˚C at argon atmosphere for 12 hours. Then reaction mixture was cooled to room temperature, diluted with water and extracted with chloroform (3×15 ml). Combined organic layers were washed with HCl solution (5% mass., 2×15 ml) and distilled water (3×15 ml). After drying over Na_2SO_4 chloroform was removed in vacuum and desired product was isolated by flesh-chromatography (SiO_2, gradient elution from $CHCl_3$ to $CHCl_3$:MeOH=100:1 (v/v)) to obtain 0.164 mg of 2 as orange solid (yeild 50%). M.p.≥231˚C (decomp.) ^{1}H NMR (400.13 MHz, DMSO-d_6, 22˚C): δ = 3.83 (s, 3H, -OCH_3), 3.90 (s, 3H, -OCH_3), 7.04 (d, 1H, *J* = 8.3, H (15)), 7.36 (dd, 1H, *J* = 8.3, *J* = 1.8, H(16)), 7.56 (d, 1H, *J* = 1.8, H(12)), 7.64 (d, 1H, *J* = 16.1, H(10)), 7.97 (dd, 1H, *J* = 8.5, *J* = 7.1, H(6)), 8.13 (d, 1H, *J* = 16.1, H(9)), 8.28 (d, 1H, *J* = 7.9, H(3)), 8.51 (d, 1H, *J* = 7.9, H(2)), 8.58 (d, 1H, *J* = 7.1, H(7)), 9.13 (d, 1H, *J* = 8.5, H(5)). ^{13}C NMR (150.93 MHz, DMSO-d_6, 21.4°C): δ = 55.39 (OCH_3(14)), 55.57 (OCH_3(13)),109.73 (C(12)), 111.45 (C(15), 116.59 (C(1)), 119.14 (C(9)), 120.15 (C(16)), 121.94 (C(8)), 123.02 (C(3)), 127.01 (C(6)), 128.84 (C(8a)), 129.20 (C(4a)),130.45 (C(11)), 132.05 (C(2)), 132.19 (C(7)), 132.43 (C(5)), 136.19 (C(10)), 142.61 (C(4)), 148.87 (C(13)), 149.80 (C(14)), 160.48 (C(8b)), 160.86 (C(8c)). EI-MS, m/z (I, %): 361, 360 (100) $[M]^+$, 329 (8), 301 (11), 286 (12), 285 (48), 273 (22), 245 (12), 213 (11), 202 (18). Elemental analysis: calculated (%) for $C_{22}H_{16}O_5$: C 73.33, H 4.48; found: C 73.36, H 4.65.

(E)-6-(3,4-dimethoxystyryl)-2-(prop-2-ynyl)-1H-benzo[de]isoquinoline-1,3(2H)-dione (4). Compound **3** 0.20 g (0.56 mmol) and 0.142 ml (2.22 mmol) of propargylamine were dissolved in 10 ml of 2-methoxyethanol. The reaction mixture was refluxed in argon atmosphere for 10h, then cooled to room temperature and diluted with water. The precipitate formed was filtered off and washed with HCl solution (5% mass.) and with water. Purification by recrystallization from methanol gave 0.19 g of product **4**, 86% yield. M. p. 180-182ºC. ^{1}H NMR (400.13 MHz, $CDCl_3$, 18˚C): δ = 2.29 (s, 1H, -CH), 4.04 (t, 3H, -OCH_3), 4.08 (t, 3H, -OCH_3), 5.06 (s, 2H, -CH_2), 7.02 (d, 1H, H(15)), 7.23-7.38 (m, 2H, H(12), H(16)), 7.39 (d, 1H, H(10), *J* = 16.0), 7.83 (d, 1H, H(9), *J* = 16.0), 7.86-7.98 (m, 1H, H(6)), 8.07 (d, 1H, H(3), *J* = 7.8), 8.62-8.88 (m, 3H, H(2), H(5), H(7)). ^{13}C NMR (150.93 MHz, CD_2Cl_2, 21.4°C): δ = 29.16 ($\underline{C}H_2CCH$), 55.81 (-OCH_3), 55.85 (-OCH_3), 69.95 (C^{IV}-CH), 78.94 (-CH), 109.46 (C(12)), 111.41 (C(15)), 120.60 (C(1)), 120.96 (C(12), C(9)), 122.68 (C(8)), 123.44 (C(3)), 126.53 (C(6)), 128.66 (C(8a)), 129.45 (C(11) or (C(4a)), 130.39 (C(5)), 131.16 (C(7)), 131.21

(C(2)), 135.39 (C(10)), 142.09 (C(4)), 149.43 (C(13) or (C(14)), 150.23 (C(13) or (C(14)), 164.04 (C(8b)), 164.30 (C(8c)). ESI-MS, m/z: 398 $[M+H]^+$.

(E)-2-((1-(2-((2-azidoethyl)disulfanyl)ethyl)-1H-1,2,3-triazole-4-yl)methyl)-6-(3,4-dimethoxystyryl)-1H-benzo[de]isoquinoline-1,3(2H)-dione (NI–S–S–N_3).

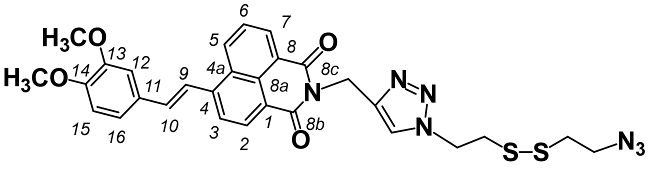

NI-S-S-N_3

To solution of compound **2** (0.102 g, 0.50 mmol) in dichloromethane (2 ml) was added 57 mg (0.17 mmol) of compound **4**, 5.2 mg CuI and 0.116 ml (0.066 mmol) of DIPEA. Reaction mixture was stirred at room temperature under argon atmosphere for 32 h. Then the solvent was removed under reduced pressure and the desired product was isolated by column chromatography (SiO_2, gradient elution from CH_2Cl_2 to CH_2Cl_2/MeOH = 20/1 (v/v)) to give 0.03 g of **NI-S-S-N3** as orange oil (yield 43%). ^{1}H NMR (400.13MHz, CD_2Cl_2, 18°C): δ = 2.82 (t, 2H, *J* = 6.6, -SC<u>H</u>$_2$CH$_2$N$_3$), 3.14 (t, 2H, *J* = 7.0, -triazole-CH$_2$C<u>H</u>$_2$S), 3.54 (t, 2H, *J* = 6.6, -SCH$_2$C<u>H</u>$_2$N$_3$), 3.89 (s, 3H, -OCH_3), 3.94 (s, 3H, -OCH_3), 4.62 (t, 2H, *J* = 7.0, -triazole-C<u>H</u>$_2$CH$_2$S), 5.44 (s, 2H, triazole-CH$_2$-N), 6.93 (d, 1H, *J* = 9.0, H(15)), 7.18-7.23 (m, 2H, H(12), H(16)), 7.33 (d, 1H, *J* = 16.0, H(10)), 7.70-7.82 (m, 3H, H(9), H(6), CH(triazole)), 7.99 (d, 1H, *J* = 7.8, H(3)), 8.55 (d, 1H, *J* = 7.8, H(2)), 8.58-8.66 (m, 2H, H(5), H(7)). ^{13}C NMR (150.93 MHz, ДМСО-*d*$_6$, 21°C): δ = 35.11 (CH_2N), 37.49 (S<u>C</u>H$_2$CH$_2$N$_3$), 37.80 (triazole-CH$_2$C<u>H</u>$_2$S), 48.78 (triazole-<u>C</u>H$_2$CH$_2$S), 49.81 (CH_2N_3), 55.81 (OCH_3), 55.85 (OCH_3), 109.46 (C(12)), 111.41 (C(15)), 120.81 (C(1)), 120.93 (C(9) or C(16)), 121.06 (C(9) or C(16)), 122.86 (C(8)), 123.43 (C(3)), 123.89 (CH-triazole), 126.52 (C(6)), 128.72 (C(8a)), 129.45 (C(4a) or C(11)), 129.59 (C(4a) or C(11)), 130.24 (C(5)), 131.09 (C(2) or C(7)), 131.13 (C(2) or C(7)), 135.31 (C(10)), 141.94 (C(4)), 143.75 (C^{IV}-triazole), 149.45 (C(13) or C(14)), 150.26 (C(13) or C(14)), 163.54 (C(8b)), 163.85(C(8c)). EI-MS, m/z (I, %): m/z 548 (23) $[M+Na]^+$.

Conjugate BChl–S–S–NI.

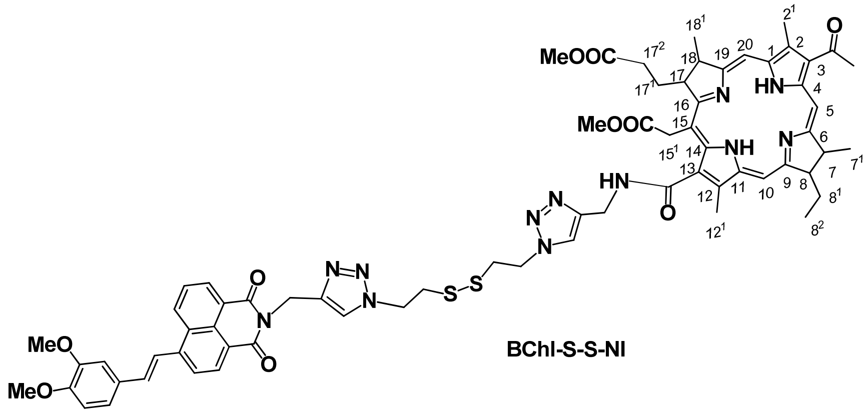

BChl-S-S-NI

In 2 ml of dichloromethane 20 mg (0.03 mmol) of **BChl** and 18 mg (0.03 mmol) of **NI–S–S–N**$_3$were dissolved. Then 50 µL (0.29 mmol) DIPEA was added and the mixture was bubbled with argon for 10 min, then 1mg (5 µmol) of CuI was added and resulting mixture was stirred for 1.5 h in the dark under inert atmosphere. The progress of the reaction was monitored by TLC. Upon completion, the mixture was deluted with 10 ml of water and

extracted with dichloromethane (4·3ml), then dried over sodium sulfate. The product was isolated as dark oil by preparative chromatography with 51% yield. ^{1}H NMR (400.13 MHz, CD_2Cl_2, 18˚C): -1.69 (s, 1H, -NH), -1.66 (s, 1H, -NH), 0.98 (t, 3H, *J* = 7.4, $CH_3(8^2)$), 1.70 (d, 3H, $CH_3(18^1)$), 1.82-1.93 (m, 4H, $H(17^1)$, $CH_3(7^1)$), 2.10-2.29 (m, 4H, $H(17^1)$, $H(17^2)$, 2×$H(8^1)$), 2.53-2.63 (m, 1H, $H(17^2)$), 3.08 (s, 3H, CH_3CO), 3.12 (s, 3H, $CH_3(12^1)$), 3.13-3.21 (m, 2H, S-$C\underline{H}_2$), 3.25 (t, 2H, *J* = 6.3, S-$C\underline{H}_2$), 3.43 (s, 3H, $CH_3(2^1)$), 3.60 (s, 3H, $COOCH_3$), 3.79 (s, 3H, $COOCH_3$), 3.92 (s, 6H, 2×OCH_3'), 4.16-4.35 (m, 4H, H(8), H(17), H(18), H(7)), 4.52 (t, 2H, *J* = 5.8, -$C\underline{H}_2$-triazole), 4.65 (t, 2H, *J* = 6.6, -$C\underline{H}_2$-triazole), 4.88-4.96 (m, 1H, $C\underline{H}_2NHCO$), 5.08-5.28 (m, 4H, $C\underline{H}_2NHCO$, $CH_2(15^1)$, CH_2-NI), 5.43-5.55 (m, 1H, CH_2-NI), 6.28 (d, 1H, *J* = 16.0, H(10′)), 6.41-6.51 (m, 2H, H(9′), H(16′)), 6.72 (s, 1H, H(12′)), 6.74 (d, 1H, *J* = 7.8, H(3′)), 6.87 (d, 1H, *J* = 7.8, H(15′)), 7.04-7.11 (m, 1H, H(6′)), 7.51-7.58 (m, 2H, H(2′), H(7′)), 7.79 (s, 1H, CH-triazole), 8.01 (d, 1H, *J* = 7.4, H(5′)), 8.06 (s, 1H, CH-triazole), 8.24 (s, 1H, H(10)), 8.66 (s, 1H, H(5)), 9.26 (s, 1H, H(20)). ^{13}C NMR (100.60 MHz, CD_2Cl_2, 18°C): δ = 11.10 ($C(8^2)$), 12.08 ($C(12^1)$), 14.00 ($C(2^1)$), 23.63 ($C(18^1)$), 23.79 ($C(7^1)$), 29.97 ($C(17^1)$), 30.22 ($C(8^1)$), 31.49 ($C(17^2)$), 33.51 ($\underline{C}H_3CO$), 35.43 ($C(15^1)$, 36.79 ($\underline{C}H_2NHCO$), 38.04 ($\underline{C}H_2$-NI), 38.92 (S-CH_2), 39.29 (S-CH_2), 47.13 (C(7) or C(18)), 48.51 (C(7) or C(18)), 49.30 (CH_2-triazole), 49.66 (CH_2-triazole), 52.02 ($COO\underline{C}H_3$), 52.76($COO\underline{C}H_3$), 53.73 (C(17)), 56.44 (2×OCH_3), 57.56 (C(8)), 96.39 (C(10)), 98.01 (C(5)), 98.51 (C(20)), 101.84 (C(14)), 109.90 (C(12′)), 111.89 (C(15′)), 115.27 (C(1′)), 117.30 (C(8′)), 120.35 (C(9′)), 121.18 (C(3′)), 122.27 (C(8a′)), 122.71 (C(16′)), 123.48 (C(4a′), C(11′)), 123.81 (CH-triazole), 124.77 (CH-triazole), 126.38 (C(6′)), 128.74 (C(3)), 129.82 (C(11)), 130.84 (C(2′), C(7′)), 131.03 (C(5′)), 131.66 (C(12)), 132.23 (C(1)), 132.53 (C(13)), 133.19 (C(2)), 133.91 (C(15)), 134.76 (C(10′)), 135.23 (C(4)), 144.20 (C(4′)), 145.75 (C^{IV}-triazole), 147.02 (C^{IV}-triazole), 150.01 (C(14′), C(13′)), 162.90 (C(19)), 163.87 (C(8c′) or C(8b′)), 164.05 (C(8c′) or C(8b′)), 167.90 (C(6)), 168.52 (C(9)), 169.59 (C(16)), 173.18 (NHCO), 173.95 ($\underline{C}OOCH_3$), 174.10 ($\underline{C}OOCH_3$), 198.54 ($\underline{C}OCH_3$). Mass (MALDI): 1280.373 (M^+).

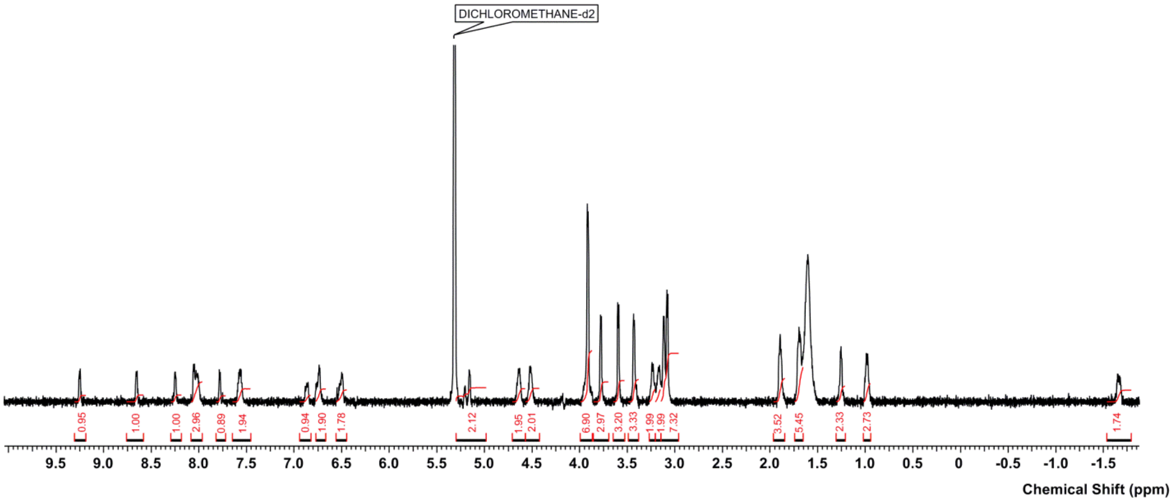

Figure S2. ^{1}H NMR spectra of BChl–S–S–NI in CD_2Cl_2 (400.13 MHz).

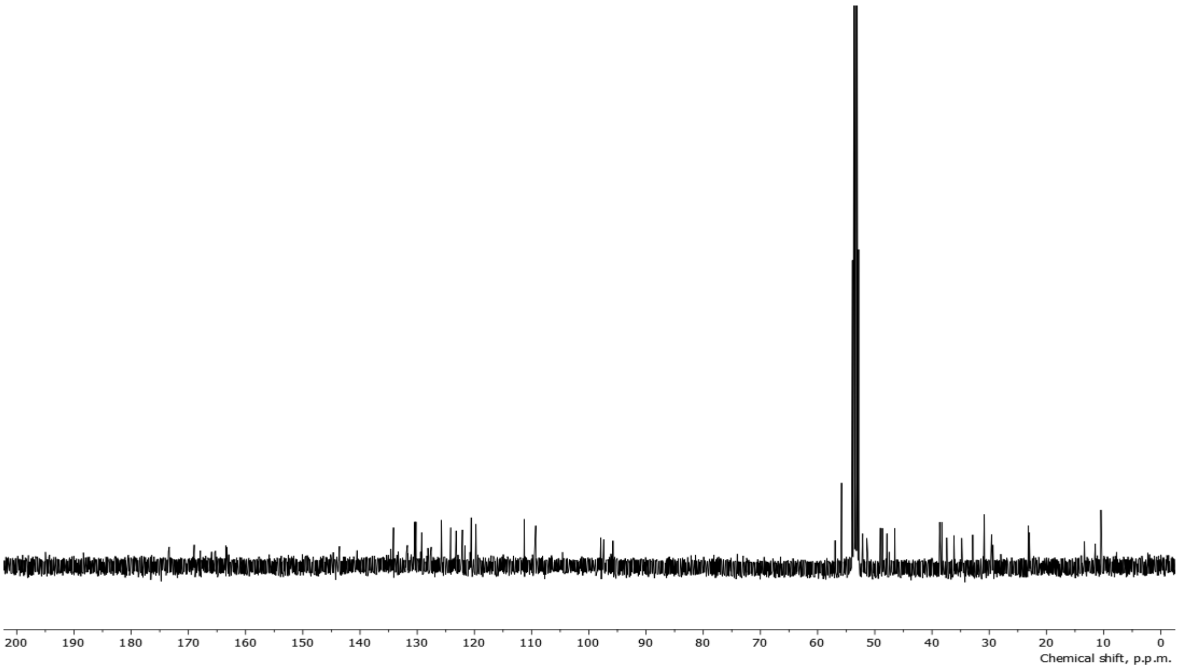

Figure S3. ^{13}C NMR spectra of BChl–S–S–NI in CD_2Cl_2 (100.60 MHz).

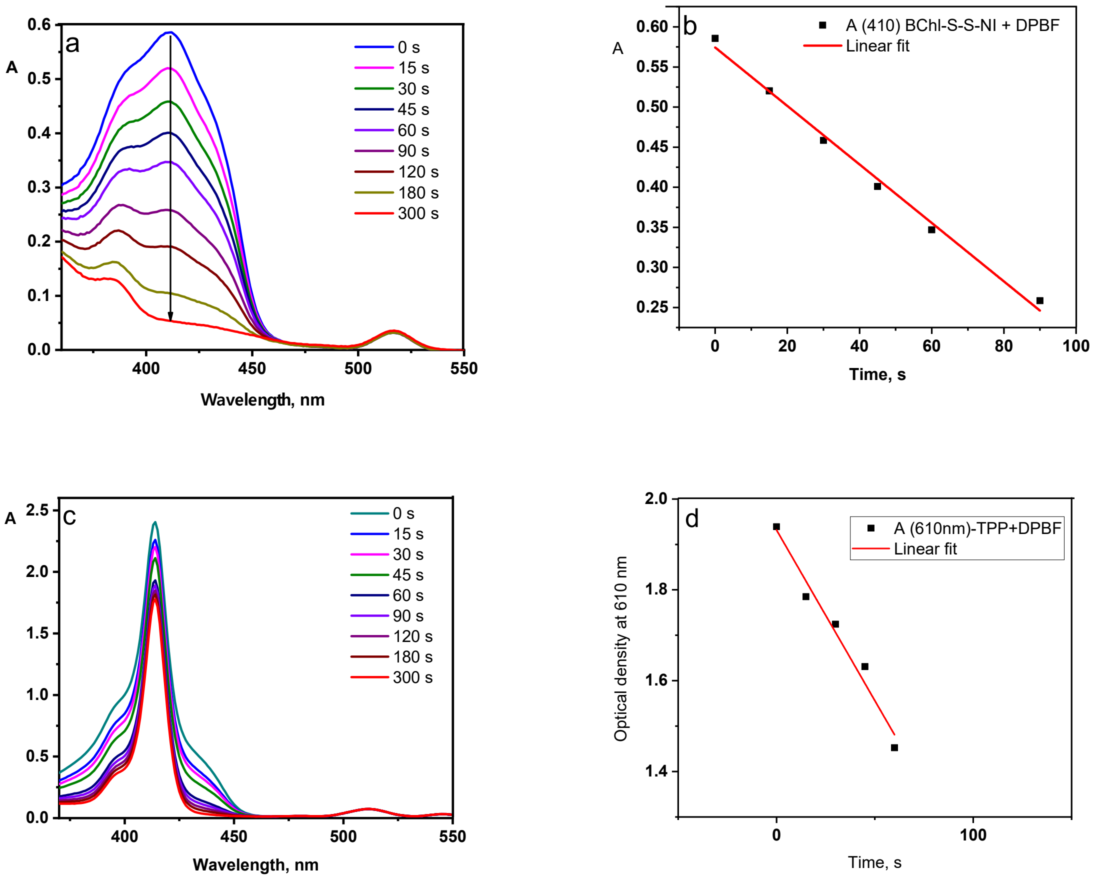

Figure S4. a, c - changes in the UV/Vis absorption spectrum of a mixed solution containing the conjugate BChl–S–S–NI ($2.6 \cdot 10^{-6}$ M, Figure.S4a) and tetraphenylporphyrin (TPP, Figure.S4c) in presence of DPBF ($4.0 \cdot 10^{-5}$ M) in acetone upon irradiation at 510 nm. b,d - the dependence of the electron density of the mixture of BChl–S–S–NI (Figure.S4b) or TPP (Figure.S4d) and DPBF at 410 nm on the irradiation time (black squares), the linearization of this function (red line) and its parameters used in calculating the quantum yield of singlet oxygen generation.

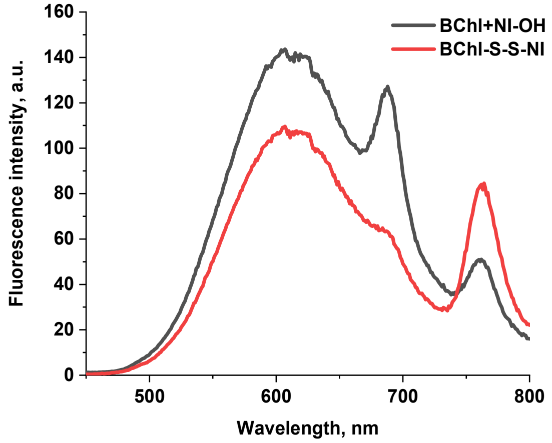

Figure S5. Fluorescence spectra of the equimolar mixture of BChl and NI–OH and BChl–S–S–NI after 21 hour of exposure in the presence of 5mM glutathione. HEPES buffer solution (0.01 M, pH 7.4) at 37°C with 1% TritonX100, excitation 420 nm.

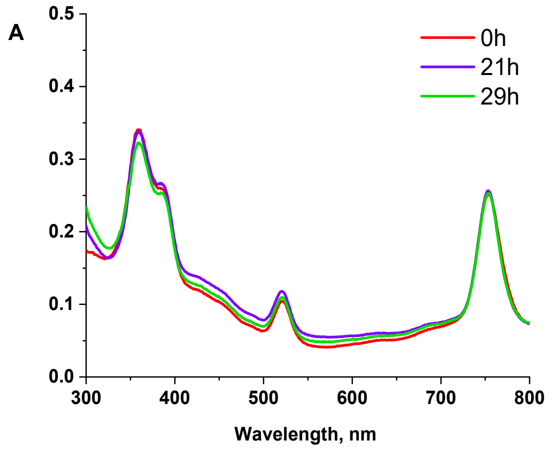

Figure S6. Absorption spectra of BChl–S–S–NI (5 μM) upon exposure with glutathione (5 mM), from 0min to 29 h. HEPES buffer solution (0.01 M, pH 7.4) at 37°C with 1% TritonX100.

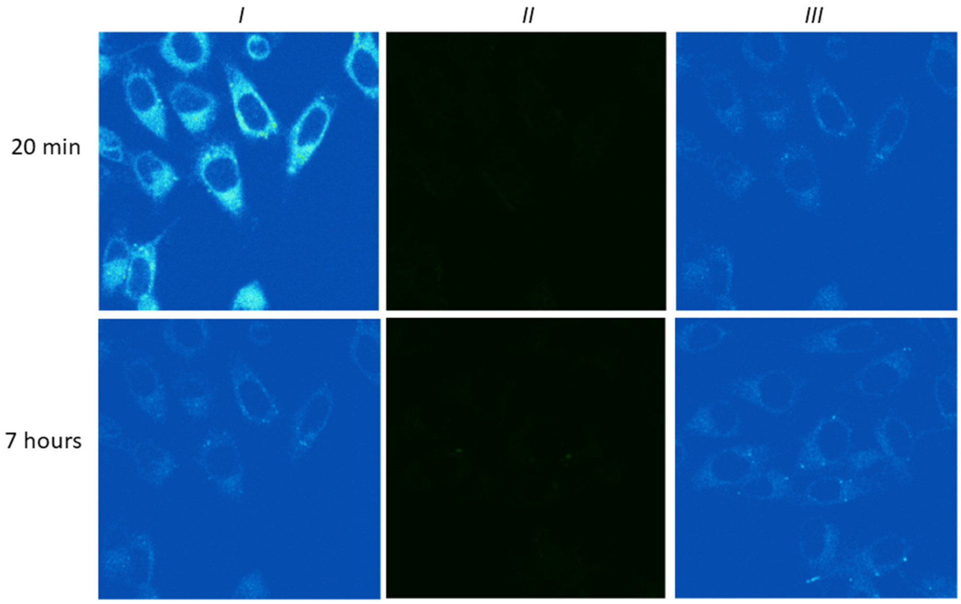

Figure S7. Confocal fluorescent images of S37 cells incubated with 8 μM BChl for 20 min (top row) or 7 h (bottom row). (Column I) Distribution of fluorescence excited at 514 nm and registered at >730 nm. (Columns II, III) Distribution of fluorescence excited at 458 nm and registered in the 570-620 nm range (column II) or at >730 nm (column III). Bar is 20 μm.

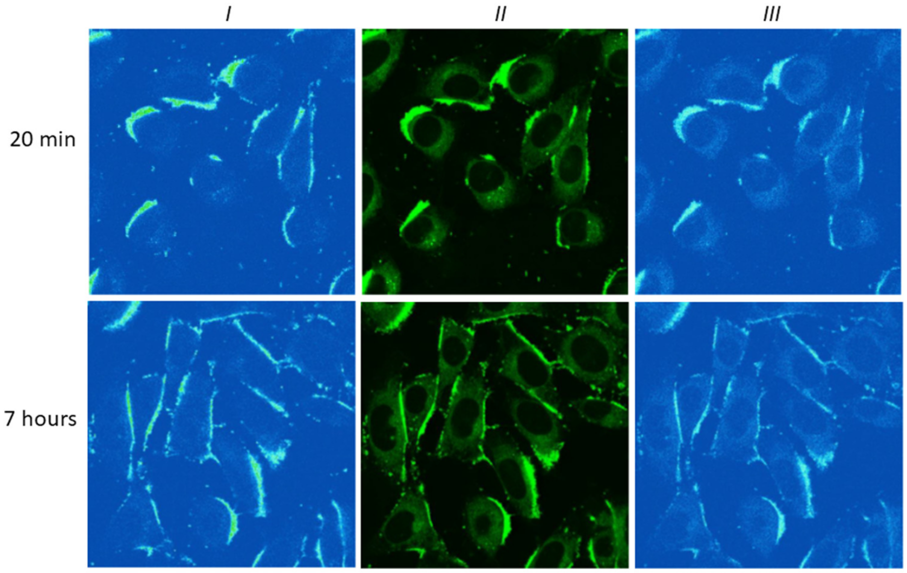

Figure S8. Confocal fluorescent images of S37 cells incubated with 8 μM NI–OH for 20 min (top row) or 7 h (bottom row). (Column I) Distribution of fluorescence excited at 514 nm and registered at >730 nm. (Columns II, III) Distribution of fluorescence excited at 458 nm and registered in the 570-620 nm range (column II) or at >730 nm (column III). Bar is 20 μm.
